# The transcriptome of Darwin’s bark spider silk glands predicts proteins contributing to dragline silk toughness

**DOI:** 10.1038/s42003-019-0496-1

**Published:** 2019-07-25

**Authors:** Jessica E. Garb, Robert A. Haney, Evelyn E. Schwager, Matjaž Gregorič, Matjaž Kuntner, Ingi Agnarsson, Todd A. Blackledge

**Affiliations:** 1 Department of Biological Sciences, University of Massachusetts Lowell, 198 Riverside Street, Olsen Hall 414, Lowell, MA 01854 USA; 2 Evolutionary Zoology Laboratory, Biological Institute Jovan Hadži ZRC SAZU, Novi trg 2, P.O. Box 306, 1001 Ljubljana, Slovenia; 3 Evolutionary Zoology Laboratory, Department of Organisms and Ecosystems Research, National Institute of Biology, Večna pot 111, 1000 Ljubljana, Slovenia; 4 Department of Biology, University of Vermont, Burlington, VT 05405 USA; 5 Integrated Bioscience Program, Department of Biology, The University of Akron, Akron, OH 44325 USA

**Keywords:** Molecular evolution, Biomechanics, Transcriptomics

## Abstract

Darwin’s bark spider (*Caerostris darwini*) produces giant orb webs from dragline silk that can be twice as tough as other silks, making it the toughest biological material. This extreme toughness comes from increased extensibility relative to other draglines. We show *C. darwini* dragline-producing major ampullate (MA) glands highly express a novel silk gene transcript (MaSp4) encoding a protein that diverges markedly from closely related proteins and contains abundant proline, known to confer silk extensibility, in a unique GPGPQ amino acid motif. This suggests *C. darwini* evolved distinct proteins that may have increased its dragline’s toughness, enabling giant webs. *Caerostris darwini’s* MA spinning ducts also appear unusually long, potentially facilitating alignment of silk proteins into extremely tough fibers. Thus, a suite of novel traits from the level of genes to spinning physiology to silk biomechanics are associated with the unique ecology of Darwin’s bark spider, presenting innovative designs for engineering biomaterials.

## Abstract (web summary)

Jessica Garb et al. report the transcriptome of the major ampullate silk gland of Darwin’s bark spider, known for its extraordinarily tough silk. They identify a novel predicted protein, MaSp4, which is highly divergent compared to related proteins in other species and has properties predicted to confer silk extensibility.

## Introduction

Spider silks are the toughest biological materials in nature due to combined strength and extensibility, leading to enormous interest in engineering silk-based biomaterials for industrial applications^1^. Among the seven silk types spun by orb-weaving spiders, dragline from major ampullate (MA) glands is the most studied for its high tensile strength and toughness that dissipates kinetic energy from flying prey in orb web radial and frame lines^2–4^. Dragline silk of Darwin’s bark spider (*Caerostris darwini*) from Madagascar can be twice as tough as any other silks measured (354 ± 93 MJ m^−3^) and 10-fold tougher than Kevlar due to characteristic spider silk strength combined with unusual extensibility (up to 91% its length)^5^. Extraordinarily tough dragline is hypothesized to be adaptive for *C. darwini* because it constructs the largest recorded orb webs, up to 2.8 m^2^, suspended by bridgelines up to 25 m across rivers, capturing prey inaccessible to most predators^5,6^. Determining the molecular basis for the toughness of *C. darwini* dragline is key for understanding both the evolutionary origins of this exceptional foraging strategy and for biomimetic research aiming to replicate the impressive properties of this ultra-tough silk.

Spider silks are formed from spidroins, a family of repetitive structural proteins exhibiting differing expression among the diverse toolkit of spider silk glands^7–9^. The amino acid motifs composing spidroin repeats are highly variable, generating the distinctive mechanical and functional properties of each silk type^9–12^. Repetitive regions are flanked by amino (N) and carboxy (C)-terminal domains critical for fiber assembly, which also serve as phylogenetic markers^13,14^. Orb-weaver dragline is primarily comprised of MA spidroins MaSp1 and MaSp2, both having repetitive regions containing poly-alanine (A_n_) amino acid motifs that form β-sheet secondary structures and contribute to tensile strength by stacking into nanocrystals^11^. MaSp2 also contains many GPGX motifs (X typically is G, S, A, or Q), which form β-turns supplying dragline extensibility^11,12^. Many GPGX, but no A_n_ motifs, are also found in the flagelliform silk spidroin Flag, the protein in the orb web’s highly elastic capture spiral, where GPGX-forming β-turns assemble into “nano-springs,” allowing up to ≥1000% reversible extensibility^10,15,16^.

The toughness of *C. darwini* dragline silk is in part due to greater extensibility relative to other species’ dragline, and Agnarsson et al.^5^ hypothesized that *C. darwini* dragline might contain novel protein secondary structures to explain its increased extensibility, and hence toughness, relative to other orb-weaver silk. Here we investigate the diversity, expression, and evolution of *C. darwini* MA gland spidroins to identify unusual sequence features that might explain the extreme extensibility and toughness of *C. darwini* dragline. In addition to typical MaSp1- and MaSp2-like transcripts in the MA silk glands, we describe, to our knowledge, a novel MaSp4a transcript that is also abundant in MA glands. However, the MaSp4a protein lacks the repetitive poly-alanine motif typical of MA spidroins and is instead dominated by the proline-rich motif GPGPQ, which may in part explain the greater extensibility and toughness of *C. darwini* dragline silk. In addition to divergent MaSp spidroins, we describe an unusually long spinning duct associated with *C. darwini* MA glands. Given that this duct is the site of spidroin alignment and intermolecular bonding to form fibers, these results suggest the hypothesis that *C. darwini* has evolved distinct molecular and physiological mechanisms for producing extremely tough dragline silk for its exceptionally large webs.

## Results

### *Caerostris darwini* expresses diverse and novel MA spidroins

To obtain *C. darwini’s* MA spidroins, we used single molecule real-time (SMRT) sequencing^17^ of MA gland expressed gene transcripts (complementary DNAs (cDNAs)) generated with the isoform sequencing (Iso-Seq) method to produce 10,666 consensus sequences. The Iso-Seq method of SMRT sequencing with Pacific Biosciences instruments generate continuous reads covering the complete length of individual cDNA molecules, overcoming the problems of assembling long, repetitive spidroins encountered with shorter reads from Illumina and Sanger sequencing (see Methods). To obtain additional expression information, a *C. darwini* MA gland transcriptome was also constructed from two Illumina RNA-sequencing (RNA-Seq) libraries producing 206,838 unique sequences. In both assemblies we surveyed spidroin diversity by clustering translated sequences containing C-terminal domains with ≥95% identity. This yielded 14 sequence groups, seven with best BLAST hits to MaSp sequences. The remainder had top BLAST hits to spidroins associated with other silk types: PySp (piriform/cementing silk spidroin), MiSp (minor ampullate silk spidroin), TuSp (tubuliform/egg-case silk spidroin), Flag (flagelliform/capture spiral silk spidroin), AcSp (aciniform/wrapping silk spidroin), and AgSp (aggregate/glue spidroin; Supplementary Data 1).

**Fig. 1:**
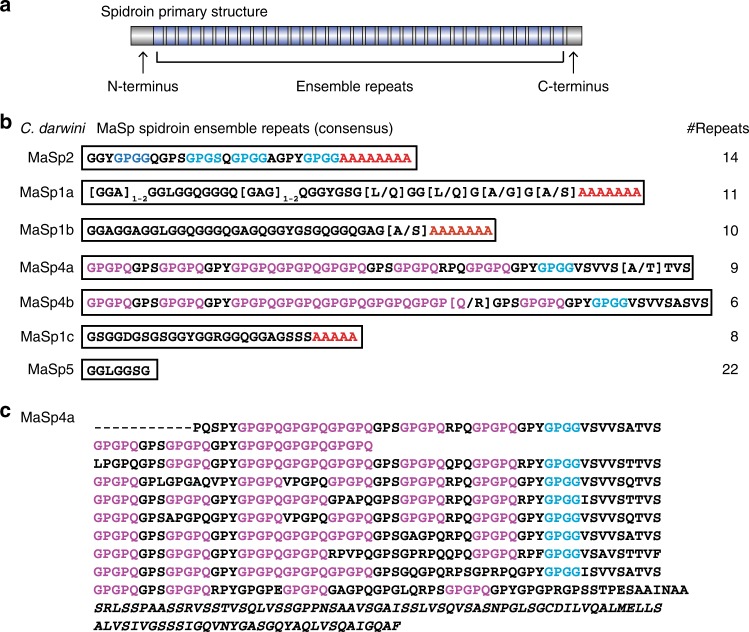
Unusual proline-rich proteins (MaSp4a and MaSp4b) among *Caerostris darwini* major ampullate Spidroin (MaSp) proteins. **a** Schematic of spider silk proteins (spidroins) composed of tandem-repeated amino acid sequences (ensemble repeats) flanked by non-repetitive amino (N)- and carboxy (C-) terminal domains. **b** *Caerostris darwini* MaSp proteins with each box presenting consensus repeat and repeat numbers determined from the longest sequence of each type (full sequences in Supplementary Data 10); poly-alanine (A_n_) motifs in red, GPGX (X = G, S, A, or Q) in blue, and GPGPQ motifs in purple. **c** MaSp4a protein aligning consecutive ensemble repeats with C-terminal domain italicized, highlighting GPGPQ and GPGX as above

We examined the longest spidroin in each C-terminal cluster and found three were most similar to MaSp1, having GGX and A_n_ motifs in repetitive sequence, whereas one was most similar to MaSp2 with combined GPGX, GGX, and A_n_ motifs (Fig. 1). However, three newly described spidroins had C-termini with top BLAST hits to MaSp1 or MaSp2, but lacked poly-alanine (A_n_) motifs. One of these, MaSp5, is mostly composed of GGX motifs. By contrast, MaSp4a and MaSp4b are strikingly unique spidroins enriched with, to our knowledge, novel GPGPQ motifs that occupy 44–52% of the repetitive region (Fig. 1c). MaSp4 is markedly different from the eight MaSps from the genome of another orb-weaver *Trichonephila clavipes*^7^ (formerly *Nephila clavipes*^18^), with GPGPQ only appearing once in *Trichonephila* MaSp-g and iterations of this motif are not seen in other species’ spidroins. Our longest MaSp4a includes nine repeats, seven of which are 63 amino acids long and contain four to six GPGPQ motifs, one GPGG motif, and one VSVVSTTVS motif (Fig. 1c). We did not identify *C. darwini* spidroins similar to MaSp3^19^, which was recently described as having MaSp terminal domains but repetitive sequence lacking polyalanine and GPGX motifs. This absence of MaSp3 may be because our data does not survey all spidroins at the genomic level (how several MaSp3 sequences were identified^19^), or it is also possible that this paralog is not present in *C. darwini* given the apparent frequency of gene duplications and losses in the spidroin family^20^.

### Composition and structure of *C. darwini* MaSps and dragline

**Fig. 2:**
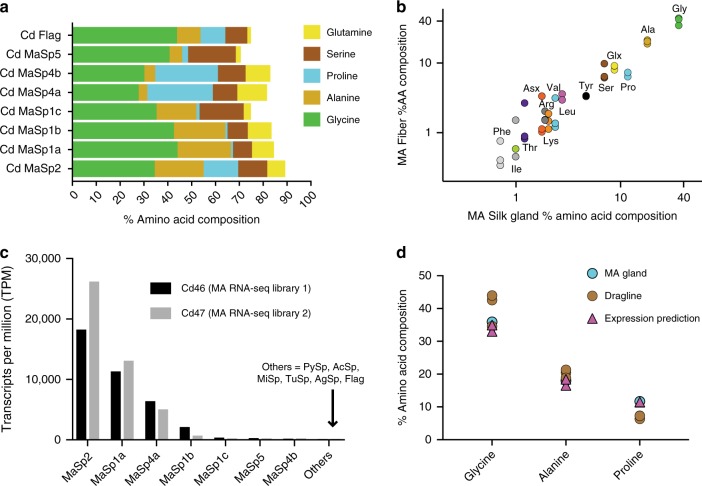
Amino acid composition and expression of *Caerostris darwini* major ampullate (MA) spidroin (MaSp) proteins consistent with MA gland and dragline fiber composition. **a** Amino acid composition of *C. darwini* MaSp and Flag proteins (percent five most abundant residues) based on translation of longest transcript for each (Supplementary Data 1, 3). **b** Percent amino acid composition of dragline (major ampullate) fibers (*n* = 3) against major ampullate gland composition (*n* = 1), five most abundant residues color coded as in part a (Glx = glutamine + glutamate). **c** Expression of spidroin transcripts in *C. darwini* major ampullate silk glands from two gland-specific RNA-sequencing (RNA-Seq) libraries. Expression measured in transcripts per million (TPM) in replicate individuals (cd46 and cd47), values listed in Supplementary Data 7. **d** Predicted glycine, alanine, and proline composition from expression data closely matched MA gland and dragline composition (symbols represent individual data points)

Typical orb-weaver dragline contains mainly MaSp1 and MaSp2, yielding a high content of glycine (34.7–42.2%), alanine (17.6–27.5%), and proline (1.7–15.7%)^21,22^ (Supplementary Data 2). Both proteins are dominated by glycine and alanine, but proline is almost exclusively found in the repetitive region of MaSp2 (9.1–16.4% vs. 0–0.5% in MaSp1, Supplementary Data 3a, b and 4a, b). Amino acid compositions of *C. darwini* MaSp1a-c and MaSp2 are similar to those in other species (Supplementary Data 3a, b– and 4a, b). By contrast, the repetitive region of *C. darwini* MaSp4 contains 31.4–32.0% proline and is deficient in alanine (2.2–2.5%; Fig. 2a). Higher proline is linked with greater silk extensibility^21,22^, and MaSp4’s ~32% proline content substantially exceeds the 10.9–16.3% proline in the Flag spidroin from elastic capture silk (Fig. 2a; Supplementary Data 4b). We note that the *C. darwini* spidroins presented here are based on partial transcripts, as is most typically obtained from spidroin cDNAs. Nevertheless, amino acid compositions inferred from translations of partial spidroins are expected to be similar to full-length proteins given that spidroins are largely composed of highly repetitive sequences.

The abundant proline of MaSp2 and Flag is associated with GPGX motifs, which form β-turns conferring extensibility to dragline and flagelliform silk^10,12^. We used the Garnier algorithm^23^ to predict secondary structures in *C. darwini* spidroins and found the highest percentage of turns assigned to MaSp4a (32.2%) and MaSp4b (31.6%), exceeding MaSp2 (17.2–26.9%) and Flag (19.3–24.7%; Supplementary Data 5). Overall proline levels in *C. darwini* dragline (6.4–7.3%; *n* = 3) and MA glands (11.7%; *n* = 1) fall within the range of other species (Fig. 2b; Supplementary Data 2) so that proline abundance per se is unlikely to explain the silk’s greater extensibility.

Instead, we propose the hypothesis that proline’s high abundance and arrangement in GPGPQ motifs in MaSp4 may increase dragline extensibility either by forming novel structural domains embedded among other MaSp proteins or by packing in more β-turns per protein monomer. Specifically, as proline is the critical residue in forming a β-turn, the additional proline per GPGPQ motif, in comparison to MaSp2 GPGX motifs (where X is rarely P), may increase the number of β-turns per motif or may produce distinct secondary structures altogether, given the steric constraints imposed by proline. These possibilities might lead to increased dragline extensibility through the addition of more β-turns within the amorphous (non-crystalline) regions or through the decreased alignment of molecules along the fiber. Testing these hypotheses would require detailed analyses of recombinantly expressed *C. darwini* MaSp4, along with MaSp1 and MaSp2, to understand how combinations of these proteins interact at the biophysical level to affect fiber mechanics.

### Proline-rich spidroin expressed in *C. darwini* MA glands

To determine which spidroins are most highly expressed in *C. darwini* MA glands and likely to have the greatest impact on dragline mechanics, we estimated Illumina-derived transcript abundance across two individual’s MA glands in TPM (transcripts per million; Supplementary Data 6 and 7). Among all spidroins and across replicates, MaSp2 had the highest expression (average TPM = 22,182.65), representing 46–56% of spidroin expression in MA glands, followed by MaSp1a (TPM = 12,223.90). MaSp4a was third most abundant (TPM = 5772.48), representing 11–16% of spidroin expression in *C. darwini* MA glands (Fig. 2c). By contrast, non-MaSp spidroins (TuSp, Flag, AcSp, PySp, MiSp, AgSp) had a combined TPM of 39.05–91.75, amounting to <0.1% of MA gland spidroin expression. The relative ratio of MaSp2, MaSp1a, and MaSp4a predict dragline containing 33.0–35.0% glycine, 16.6–18.4% alanine, and 11.4–11.5% proline. These values closely match our composition values of *C. darwini* dragline (34.6–44.0% glycine, 18.9–21.3% alanine, 6.4–7.3% proline; *n* = 3) and MA glands (36.0% glycine, 18.0% alanine, 11.7% proline; *n* = 1; Fig. 2d), consistent with spidroin transcript abundance positively correlating with dragline incorporation. Thus, high expression of the proline-rich MaSp4a in MA glands supports its functional role in *C. darwini* dragline mechanics.

### Proline-rich spidroin closely related to typical MaSps

**Fig. 3:**
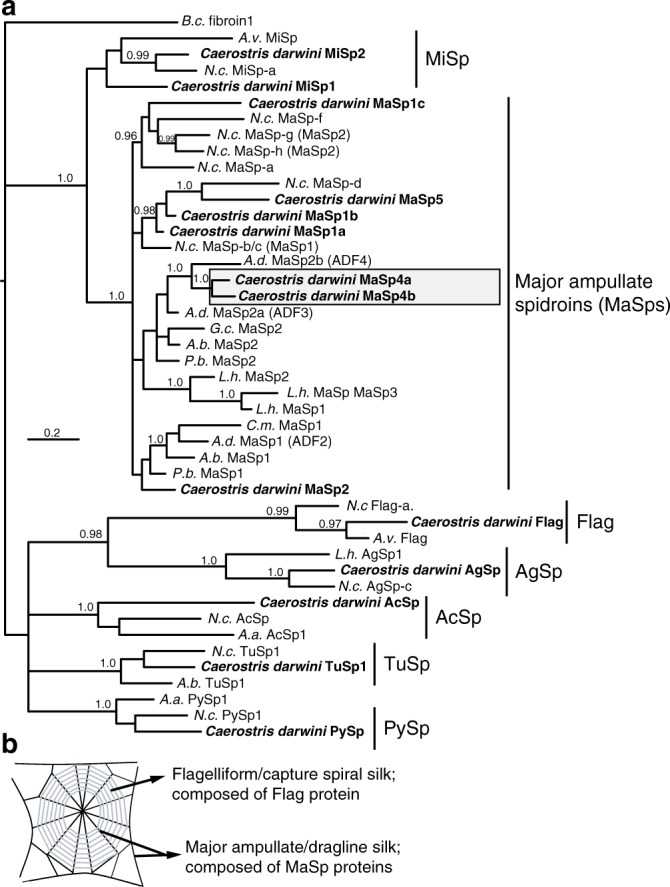
Spidroin (silk protein) carboxy (C)-terminal phylogenetic tree nests GPGPQ-rich *Caerostris darwini* proteins in major ampullate spidroin (MaSp) clade. **a** Tree is Bayesian 50% majority rule consensus of post-burn-in trees. Sequences reported in this study from *C. darwini* in bold text. Support values at nodes are clade posterior probability values where ≥0.95. Other clades of functionally assigned silk proteins highlighted as follows: PySp = piriform (attachment) silk protein; TuSp = tubuliform (egg-case) silk protein; MiSp = minor ampullate (scaffolding/bridge line) silk protein; Flag = flagelliform (capture spiral) silk protein; AcSp = aciniform (prey-wrapping) silk protein; AgSp = aggregate (glue) protein. Accession numbers in Supplementary Data 8. Species abbreviations as follows: *N.c*. = *Trichonephila clavipes*; *B.c*. = *Bothriocyrtum californicum; A.v.* *=* *Araneus ventricosus; L.h.* *=* *Latrodectus hesperus; A.b* *=* *Argiope bruennichi; A.a.* *=* *Argiope argentata; A.d.* *=* *Araneus diadematus; G. c.* *=* *Gasteracantha mammosa; C. m.* *=* *Cyrtophora moluccensis; P.b.* *=* *Parawixia bistriata*. **b** Illustration of orb web indicating position and function of MaSp and Flag proteins

To investigate *C. darwini* dragline evolution, we reconstructed spidroin phylogenetic relationships. MaSp4a and MaSp4b are firmly nested in the dragline spidroin (MaSp) C-terminal clade and appear most closely related to MaSp2 from the confamilial orb-weaver *Araneus diadematus*, with their C-termini sharing 68–69% nucleotide identity (Fig. 3, Supplementary Data 8). The assemblies also included seven spidroin N-termini from MaSp4a, MaSp4b, MaSp2, PySp, MaSp1 variants and MaSp5 (Supplementary Data 1). Their relationships similarly showed MaSp4a and MaSp4b within the MaSp N-terminal clade, but closest to *Argiope* and *C. darwini* MaSp2 (Supplementary Fig. 1). MaSp1 and MaSp2 do not form reciprocally monophyletic clades, previously attributed to intergenic concerted evolution and selection to homogenize co-expressed termini^24^. Nevertheless, our results imply the derivation of MaSp4 from a *MaSp2* gene, consistent with the GPG-rich nature of both. Consequently, while MaSp4 retains terminal domains highly similar to typical dragline proteins, its repetitive structural sequence has substantially increased in proline suggesting its adaptive evolution to support *C. darwini’s* giant webs.

### *Caerostris darwini* spinning duct may contribute to silk toughness

**Fig. 4:**
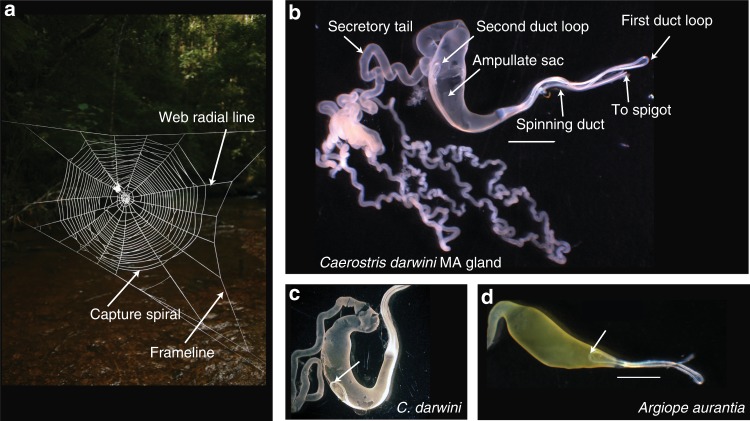
Darwin’s bark spider (*Caerostris darwini*) spins largest orb webs using silk glands with unusually long spinning ducts. **a** Orb-web of *C. darwini* illustrating major web elements: web frameline and radial lines composed of dragline (major ampullate (MA)) silk, and capture spiral composed of flagelliform silk fibers coated with aggregrate silk glue. (**b**) *Caerostris darwini* MA silk gland showing region of silk protein secretion (tail), silk solution storage sac, and fiber synthesis site (S-shaped spinning duct), indicating two loops joining three duct “limbs.” Another *C. darwini* individual (**c**) and MA gland of *Argiope aurantia* (**d**), with arrows pointing to second loop of spinning duct for comparison. Scale bars in **b**, **d** = 2 mm

Orb-weaver MA glands are subdivided into discrete sections: (1) the tail for protein secretion, (2) the ampullate sac storing liquid silk and (3) an S-shaped spinning duct for fiber assembly, terminating in an external spigot (Fig. 4)^25,26^. *Caerostris darwini’s* MA spinning ducts are unusually long with the loop connecting limbs 2 and 3 extending to the ampullate sac midpoint or beyond (average duct length along ampullate sac = 4.35 mm ± SD 0.78; *n* = 7; Fig. 4b, c). In other species examined for this study or from the literature, this duct loop does not extend further than the distal portion of the ampullate sac (e.g., average duct length along sac = 1.08 mm ± SD 0.18; *n* = 4, in *Argiope aurantia;* Fig. 4d, Supplementary Fig. 2)^25–27^. The average length of the *C. darwini* MA duct (33.64 mm ± SD 2.50) exceeds the length in *Trichonephila clavipes* (25.5 mm ± SD 4.8; *n* = 3; unpaired *t* test, *t* = 3.6495; *P* = 0.0065) and *A. aurantia* (16.3 mm ± SD 1.02; *t* = 13.0220; *P* ≤ 0.0001). Moreover, the average duct length to ampullate sac length ratio in *C. darwini* (4.26 ± SD 0.54) is 1.5–1.8× greater than in *A. aurantia* and *N. clavipes*.

The MA duct transforms liquid silk protein dope into a fiber through ion exchange, water removal, and decreasing pH along the duct^14,26,28–36^. These changes and increasing shear forces align spidroin monomers to form intermolecular secondary structures that determine silk mechanical performance (e.g. β-sheets providing strength)^31,36–40^. Extensibility and strength normally tradeoff, but *C. darwini* dragline has increased extensibility without reduced strength^5^.

We hypothesize that the lengthened spinning duct of *C. darwini’s* MA gland may facilitate alignment of spidroins to maintain dragline tensile strength (potentially by maintaining β-sheet formation) as MaSp4’s GPGPQ-containing motifs introduce increased extensibility. The longer duct may enable this by allowing the dragline to form over a longer period. X-ray diffraction by Madurga et al.^41^ showed that *C. darwini* dragline has 14% crystallinity (β-sheet structures), which was higher than the 7% crystallinity of *Argiope aurantia* dragline reported in that study, but is within ranges reported from *Trichonephila clavipes* dragline (10–28% crystallinity) from other studies^42,43^. Accordingly, the lengthened MA duct of *C. darwini* may maintain dragline crystallinity, and hence strength, within ranges observed from other orb weavers, despite increased extensibility. This hypothesis should be tested in the future by investigating biochemical and physical processes along *C. darwini’*s spinning duct.

### Evolutionary implications and biomimetic applications

Thus, in addition to MaSp2 and MaSp1, *C. darwini* MA glands highly express MaSp4a transcripts, which encode a silk protein dominated by novel GPGPQ motifs. If these motifs form β-turns similar to GPGX motifs as suggested by Garnier analysis, this would introduce more secondary structures resembling the nano-springs of flagelliform silk by which *C. darwini* dragline could achieve greater toughness through increased extensibility. That the GPGPQ motifs of MaSp4a and MaSp4b appear restricted to *C. darwini* suggests a recent origin of these proteins within the genus from *MaSp2* genes, consistent with selection for tough and extensible silk to support enormous orb webs. *Caerostris darwini’s* lengthened MA gland’s spinning duct may also contribute to assembly of especially tough dragline. Hence, a suite of traits from genes to physiology likely coevolved with the unique web architecture and ecology of *C. darwini*.

We anticipate these findings will be leveraged to produce silk-based materials mimicking the extraordinary toughness of *C. darwini* dragline. Such work could express *C. darwini* dragline spidroins in varying proportions, or engineer chimeric spidroins for biomaterials with enhanced functional properties^1^. An important open question is how spinning duct length shapes material properties. Accordingly, this study reinforces the importance of evolutionary comparative studies for discovering biotechnology opportunities.

## Methods

### Sequencing of MA gland expression libraries

Major Ampullate (MA) glands were dissected from *C. darwini* females reared by MK and MG in the laboratory, stemming from females collected in Andasibe-Mantadia National Park (around 18.94760°S, 48.41972°E at 960 m elev.), Toamasina Province, eastern Madagascar in 2012 and additional dissections were collected from specimens collected at the same locality in December 2017 (permit numbers 042N_EA04/MG12, 090/12/MEF/SG/DGF/DCB.SAP/SCB, 315N_EA12/MG17, and 280/17/MEEF/SG/DGF/DSAP/SCB, issued by Secretariat General, Direction des forets, Direction de la conservation de la biodiversite et du systeme des aires protegees). Major ampullate glands from seven *C. darwini* females were imaged with a dissecting microscope using Zeiss 2.3, along with MA glands from four female *Argiope aurantia* and three female *Trichonephila clavipes*. Duct and ampullate length were measured with ImageJ 1.50i (https://imagej.nih.gov/ij/). RNA was extracted from MA glands (one individual per extraction) by homogenization in TriZol and cleanup using Qiagen’s RNeasy kit, and removal of DNA. Using one *C. darwini* MA gland RNA extraction (cd46), cDNA was synthesized at the UMass Medical School’s Deep Sequencing Core (UMMS-DSC) using the Iso-Seq protocol^17^ (Pacific Bioscience Inc., Menlo Park, CA, USA). cDNA was fractionated into two size distributions. The larger fraction >1.2 kb was used to construct a SMRTBell^TM^ library, which was sequenced on three SMRTCells^TM^ on a PacBio RS II instrument with 120-min movies. RNA from the MA silk glands of two individuals (cd46 and cd47) was submitted to the UMMS-DSC, where cDNA was synthesized separately for each individual using the creator SMARTer method (Takara Bio USA), and fragmented to 650 bp prior to Illumina RNA-Seq library construction. The two MA gland RNA-Seq libraries were sequenced on three separate MiSeq instrument runs, sequencing 300 bp paired-end reads. Illumina adapters and SMART oligos (Supplementary Data 9) used in cDNA synthesis were trimmed from reads using CUTADAPT 1.14^44^, which was also used for quality trimming.

### Assembly of transcriptomes

Data from the SMRT sequencing cells were processed with Pacific Biosciences’ Iso-Seq1 pipeline (part of the SMRT Analysis 2.3 p5 pipeline), where the RS_IsoSeq Classify script was used to identify non-chimeric full-length transcripts (i.e., sequences containing 5′–3′ primers and poly A tails), which were used as input to the Cluster script to collapse highly similar sequences into non-redundant consensus isoforms using the ICE algorithm, followed by use of the QUIVER algorithm to “polish” isoforms with highly similar but non-full length transcripts corresponding to each full-length isoform^17,45^. Given this pipeline separates partial cDNAs of the same transcript only differing in length (because they are interpreted as full length), we further clustered tBLASTn hits to spidroin terminal domain queries using CD-HIT 4.6.4^46^ at ≥95% nucleotide identity across their full length, selecting the longest sequence per cluster for analyses. A separate de novo transcriptome from all Illumina data was generated from 14.3 million reads using Trinity 2.0.6^47^. Review by NCBI’s TSA submission pipeline identified that 0.13% of Trinity contigs represented likely contaminant sequences, which were removed from the assembly. BUSCO 2.0 (ref. ^48^) was used to evaluate the transcriptome by assessing the presence and length of conserved single-copy orthologs from arthropod species. Assembled sequences were subject to BLASTx searches against NCBI’s nr database using Diamond 0.8.23^49^, retaining hits with *e*-scores ≤e − 0.5. Translations were produced based on the frame of significant BLAST hits, or the longest open reading frame in the absence of a BLAST hit.

### Spidroin characterization

Spidroin sequences in transcriptome assemblies were identified using tBLASTn with known spidroin N- and C-terminal domain protein sequences as queries. getORF of EMBOSS 6.6.0 (http://emboss.sourceforge.net/apps/cvs/emboss/apps/getorf.html) was used to translate spidroin transcripts, and the longest translation in the frame of the BLAST hit was identified. CDhit^46^ was used to cluster translated spidroins into groups with full-length, identical terminal domains; manual inspection of all spidroin BLAST hits identified additional sequences containing complete termini, all of which were clustered into groups sharing ≥95% amino acid identity across the terminal domain. For each spidroin sequence cluster we characterized the repetitive structure of the longest sequence based on previously defined spidroin motifs^9,13,50^. Larger iterated (“ensemble”) repeats composed of combinations of these motifs were defined by aligning highly similar consecutive sequence within proteins using MUSCLE v. 3.8.31^51^, and computing a consensus reporting the modal residue for each position.

Sequences were designated as MaSp proteins if terminal domains were most closely related to previously defined MaSp termini. The MaSp1 or MaSp2 designation was based on the presence of amino acid motif combinations in the repetitive region previously defined as characteristic for those proteins^9,50^. As the recently described MaSp3^19^ was not identified among *C. darwini* transcripts, nomenclature for MaSp sequences newly described here were named MaSp4a, MaSp4b, and MaSp5, where MaSp4a and MaSp4b may represent alleles of the same protein or closely related protein paralogs. Protein secondary structure (e.g., percent helices, sheets, and/or turns) was bioinformatically predicted with the garnier EMBOSS plugin in Geneious 9.1.8^52^.

### Expression analyses

Trimmed Illumina reads from the two MA gland RNA-Seq libraries were used to estimate abundance of all Trinity assembled transcripts in TPM using Salmon v0.8.2 in quasi-mapping mode^53^ (Supplementary Data 6). Determining spidroin expression is challenging because of fragmented transcripts and incorrect mapping of repetitive regions, especially given Trinity-assembled spidroin transcripts typically contain differing lengths of repetitive sequence^20,54^. Thus, in addition to estimating TPM for all assembled Trinity-derived transcripts, we also reduced sequences in the Illumina assembly containing identical spidroin C-termini to a single representative, trimmed to 500 bp including the non-repetitive termini. TPM was re-estimated for all transcripts in this revised assembly using Salmon v0.8.2^53^ and aggregating TPM for spidroin C-termini if they shared ≥95% identity at the amino acid level (Supplementary Data 7).

### Spidroin phylogenetic analyses

Spidroin terminal domain sequences (see Supplementary Data 8 for Accession numbers) were used in phylogenetic analyses^13^ along with *C. darwini* sequences sampling C-termini representing gland-associated spidroins (TuSp1, MiSp, Flag, AcSp1, PySp1, AgSp) having linked N-terminal domains from different araneoid species, and including a greater sampling of MaSp C-termini from the family Araneidae to which *Caerostris* is classified. N-terminal phylogenetic analyses included sequences linked to C-termini used in the aforementioned C-terminal phylogenetic analysis. Sequences were aligned with MUSCLE 3.8.31^51^. Bayesian phylogenetic trees were generated from amino acid alignments using Mr. Bayes v. 3.2.6^55^. Markov Chain Monte Carlo sampling with Mr. Bayes v. 3.2.6 was run with default priors, but implementing a mixed amino acid model for 5 × 10^6^ generations plus gamma distribution, using three heated chains and one cold chain. Consensus Bayesian trees were computed from post burn-in trees (discarding the first 25%), and rooted using a mygalomorph spidroin (*B.c*. fibroin 1).

### Silk fiber and gland protein analyses

Dragline fibers were collected from forcibly silked *C. darwini* females. Three samples of spun dragline from three individuals and one pair of *C. darwini* MA glands from a single individual were sent to the UC Davis Molecular Structure Facility, and hydrolyzed with 6 N HCl for 24 h at 110 °C. This was followed by ion-exchange chromatography using an L-8800 Hitachi analyzer coupled to a post-column ninhydrin reaction system to separate and detect amino acids. One dragline sample was also run on a L-8900 Hitachi analyzer using a lithium citrate buffer to detect hydroxyproline. Results were used to compute percent molarity of amino acids in samples. Amino acid compositions of spidroin sequences were determined with ProtParam (http://web.expasy.org/protparam).

### Statistics and reproducibility

Measurement averages for silk glands are presented as mean ± standard deviation, with the number of independent biological replicates (different individuals) reported in the main text. Statistical analyses comparing differences of means were conducted with two-tailed unpaired *t* tests. Transcript expression measurements using RNA-Seq data were repeated with MA glands from two individual adult females.

### Reporting summary

Further information on research design is available in the Nature Research Reporting Summary linked to this article.

## Supplementary information

Supplementary Information

Supplementary Data 1-9

Supplementary Data 10

Description of additional supplementary items

Reporting Summary

## Data Availability

Sequence data from this study is available at NCBI’s SRA database under the accession submissions SRR7499252, SRR7499250, and SRR7499251. Assembled transcriptomes are available at NCBI’s TSA database under accession numbers GGUO00000000 and GGTX00000000. Raw data associated with Figs. 1–3 are in the Supplementary Data 1–9 and Supplementary Data 10 files.
