# Supplementary material for: The transcriptome of Darwin’s bark spider silk glands predicts proteins contributing to dragline silk toughness
Source: Commun Biol. 2019 Jul 25;2:275. doi: 10.1038/s42003-019-0496-1 (PMC6658490; doi:10.1038/s42003-019-0496-1)
Supplement: Supplementary file 1 — Supplementary Information [file 42003_2019_496_MOESM1_ESM.pdf]

Supplementary Figures

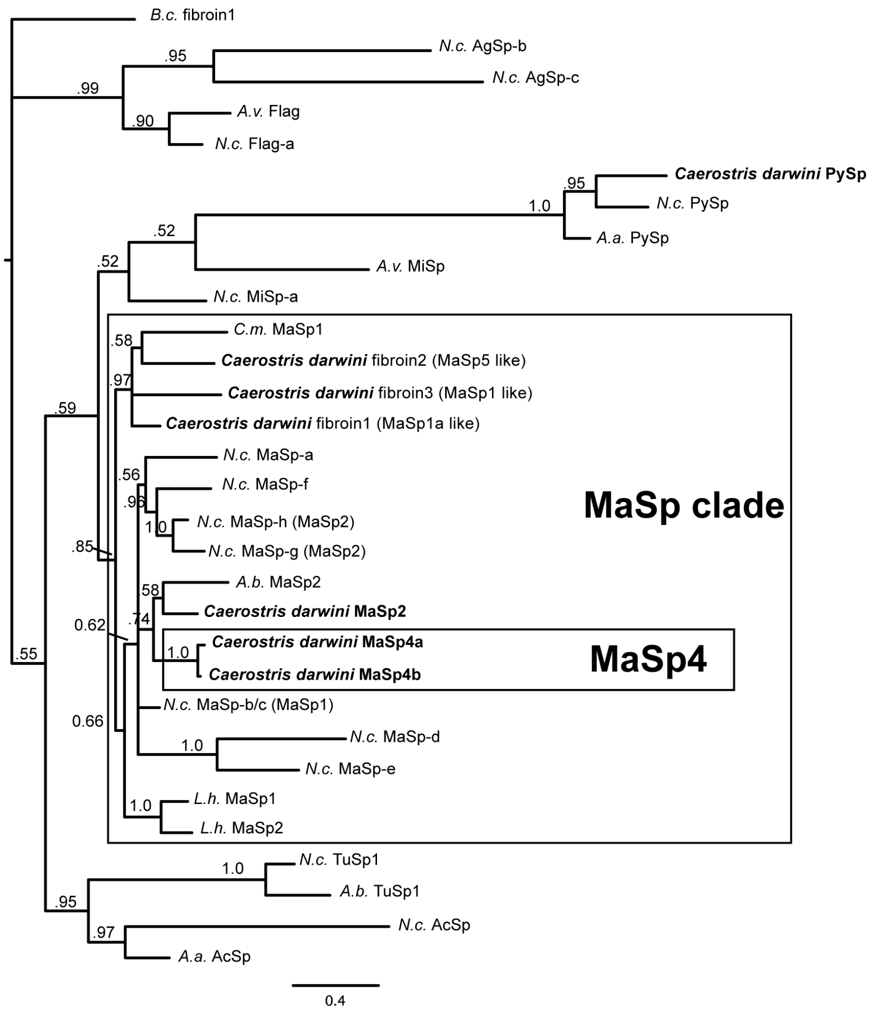

Supplementary Figure 1. Spidroin Amino (N)-terminal phylogenetic tree. 50% majority rule Bayesian consensus of amino acid alignment. Sequences from this study indicated in bold text. Support values at nodes are clade posterior probability values. Spidroin names follows: MaSp= Major ampullate (web radii/frame & dragline) silk protein; PySp=piriform (attachment) silk protein; TuSp = tubuliform (egg-case) silk protein; MiSp=minor ampullate (scaffolding/bridge line) silk protein; Flag= flagelliform (capture spiral) silk protein; AcSp = aciniform (prey-wrapping) silk protein; AgSp=aggregate = glue protein. Accession numbers in Supplementary Table 8. Species abbreviations: *N.c. = Trichonephila clavipes; B.c.= Bothriocyrtum californicum; A.v.= Araneus ventricosus; L.h.=Latrodectus hesperus; A.b= Argiope bruennichi; A.a.= Argiope argentata; A.d.= Araneus diadematus; G. c.= Gasteracantha mammosa; C. m.= Cyrtophora moluccensis; P.b.= Parawixia bistriata*

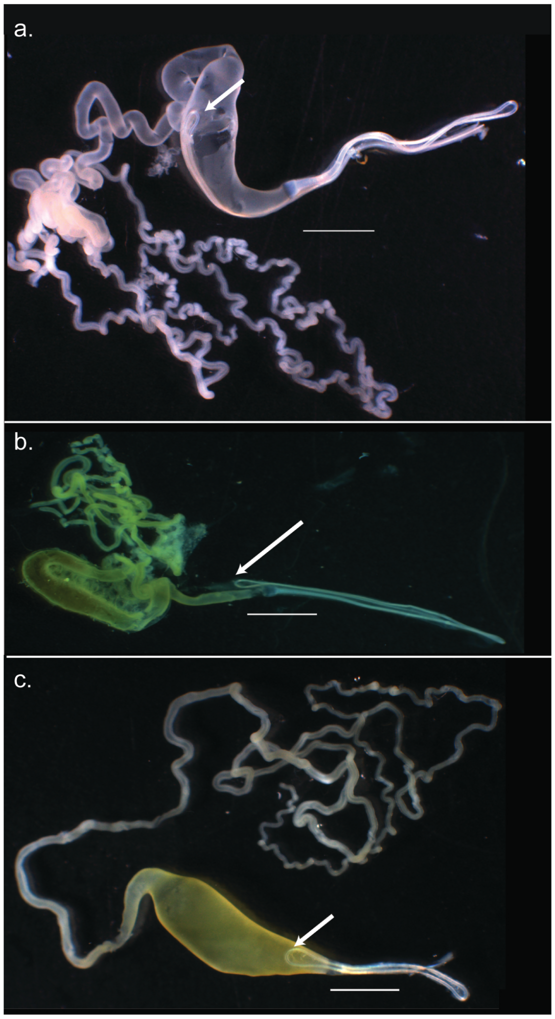

Supplementary Figure 2. (a) *Caerostris darwini* major ampullate silk gland in comparison to major ampullate silk gland from *Trichonephila clavipes* (b) and *Argiope aurantia* (c). Arrows point to loop joining limb 2 and 3 of S-shaped spinning duct. All scale bars indicate 2 mm.

Supplementary Notes

Additional description of the *C. darwini* Major Ampullate Gland Transcriptomes

SMRT sequencing of *C. darwini* major ampullate cDNA using the Iso-Seq method initially produced 93,474 non-chimeric single molecule sequences that assembled into the 10,666 Quiver-polished high quality consensus isoforms. The 10,666 Quiver-polished sequences were compiled into a BLAST database and were separately queried with known spidroin N- and C-terminal domains from functionally distinct and distantly related spidroins (e.g., MaSps, PySp, Flag, AgSp, TuSp, AcSp, MiSp, Bc fibroin 1, etc) using tBLASTn, retaining hits with e-scores ≤e-0.5. This recovered 519 C-terminal containing sequences and 61 N-terminal containing sequences. These resulting sequences were further clustered at ≥95% nucleotide identity across their full-length using CD-HIT, selecting the longest sequence per cluster for analyses, resulting in 208 C-terminal and 42 N-terminal containing sequences.

The 206,838 Illumina *de novo* assembled transcripts were used to make a BLAST database which was similarly queried with tBLASTn with the same spidroin N- and C-terminal domains used to query the Iso-Seq transcripts. Of the 206,838 sequences, 175 had tBLASTn hits to spidroin C-termini and 25 had BLAST hits to N-termini. BUSCO analyses of the Trinity transcriptome identified 92.8% of 1066 conserved arthropod benchmarking proteins that were complete, another 3.5% were present but fragmented, and 3.7% were missing.

From identified spidroins found among both the Iso-Seq/Pac Bio derived sequences and the Illumina derived sequences, translated C-terminal containing spidroins with complete C-termini were clustered if they had ≥95% amino acid identity across their termini. For the Iso-Seq

assembly C-terminal tBLASTn queries this yielded eight sequence groups, seven having best BLASTx hits to MaSp1 or MaSp2, and the eight matching the piriform/cementing silk spidroin PySp (Supplementary Data 1). The Trinity assembly tBLASTn searches recovered spidroins with these same C-terminal domains and six additional groups with top BLASTx hits to MiSp (minor ampullate silk spidroins), TuSp (tubuliform/egg-case silk spidroin), Flag (flagelliform- capture spiral silk spidroin), AcSp (aciniform wrapping silk spidroin) and AgSp (aggregate glue spidroin) (Supplementary Data 1).

Because of their highly repetitive structure and long length, the overwhelming majority of published spidroin cDNAs are partial transcripts containing some repetitive region attached to the C-terminal domain. Similarly, our spidroin transcripts and their encoding proteins are predominantly partial length, thus we chose the longest sequence in each spidroin C-terminal cluster as the best representative to analyze in greater detail. The repetitive nature of spidroins means that properties of the full-length sequence, such as amino acid composition and secondary structural predictions, may be reasonably well predicted from partial sequences. The longest sequence we report per cluster, and thus used for amino acid composition and secondary structural predictions are listed in Supplementary Data 1. The longest C-terminal containing spidroin in our Iso-Seq assembly was 2132 bp (Supplementary Data 1), whereas C-terminal containing spidroins reconstructed with Illumina reads reached a maximal length of 1990 bp.

Within some Iso-Seq spidroin sequence clusters collected from the single individual, there were multiple sequences with identical C-termini (e.g., 70 sequences with identical MaSp2 C-termini) exhibiting some variation in their otherwise similar repetitive regions relative to one another due

to insertion/deletions of amino acids as well as substitutions. These variants within clusters may represent different alleles, alternative transcripts of the same loci, potentially more than one locus, and/or transcripts of the same allele containing SMRT sequencing errors. Among these variants we note the presence of multiple short "isoforms" of MaSp2, MaSp1a, MaSp4a and MaSp4b within their respective clusters that include both N- and C-termini separated by repetitive sequence, the full length of which are shorter than the longest sequence containing the identical C-terminus attached to repetitive sequence, which are partial transcripts missing the N-terminal domain. These isoforms containing N- and C-termini may be genuine transcripts or artifacts generated during Iso-Seq cDNA library construction via PCR (e.g., due to intramolecular recombination). If genuine these would include examples of full-length spidroins, although conservatively we suggest these may be PCR artifacts.

Spidroins with connected N- and C-termini allowed us to putatively assign N-terminal sequences to some MaSp C-terminal clusters. From the two assemblies we identified seven unique *C. darwini* spidroin N-termini, three of which were connected to C-termini corresponding to MaSp4a, MaSp4b, MaSp2 in shorter variants (Supplementary Data 1). Six N-termini had BLASTx hits to either MaSp1a or MaSp1b from *Trichonephila clavipes*, while the seventh BLAST to PySp. Of the MaSp N-termini the three containing sequences not linked to C-termini had repeat motifs suggesting they may represent different MaSp1-like sequences and MaSp5 (Supplementary Data 1; Supplementary Notes).

<u>Additional description of MaSp4 features</u>

The GPGPQ motif occupies 44% of MaSp4a's repetitive region, with VSVVSQTVS motifs being 10%, and the remainder composed of GPS, GPY and GPGG motifs. MaSp4b has a highly similar structure but with repeats of 65 aa and the GPGPQ motif occupying 52% of the sequence. MaSp4a and MaSp4b share 84% amino acid identity across their C-termini suggesting they may represent the products of two closely related loci or it is possible they may represent highly divergent alleles.

The Garnier algorithm used to predict secondary structures in *C. darwini* MaSp proteins found the highest percentage of turns assigned to MaSp4a (32.2%) and MaSp4b (31.6%). In MaSp4a and MaSp4b turns were assigned to recurring PG and PY, while the less abundant VSVVSQTVS motifs are assigned to beta-sheets, suggesting they may be structurally similar to polyalanine (A_n) motifs. BLASTp queries solely using the MaSp4a repetitive region retrieved best hits to fungal trans-sialidase (OAA37422.1; 6e-38) and collagen triple-helix containing bacterial proteins (e.g, SNS09482; 9e-25), the latter of which have iterated GPQGP motifs, similar to GPGPQ motifs in MaSp4a. The collagen triple helix (type-2 helix) is composed of repeated GLY-X-Y, where X and Y are frequently proline or hydroxyproline, and provides mechanical strength to animal tissues, and in bacteria can function in biofilm formation[1]. Our composition analysis found only 0.2% hydroxyproline in *C. darwini* dragline (Supplementary Data 2).

<u>Additional notes on spidroin expression and dragline composition</u>

Past work suggests that MaSp1 is more abundant than MaSp2 in *Trichonephila clavipes* dragline in a 3:2 ratio [2,3] and *Latrodectus hesperus* MA gland expression suggests a 2.5-3:1 ratio of MaSp1:MaSp2 [4,5]. Our *C. darwini* major ampullate gland expression data (Supplementary Data

7) suggests an average ratio of 3.9: 2.1: 1 for MaSp2: MaSp1a: MaSp4a, with MaSp2 having the greatest expression in contrast to the *Trichonephila* and *Latrodectus* dragline estimates. High expression of MaSp2 and MaSp4a in *C. darwini* major ampullate glands would also be consistent with the higher proline content of *C. darwini* dragline (6.9%) relative to values reported for *N. clavipes* (4.3%) and *L. hesperus* (2.5%; Supplementary Data 2).

Supplementary References

1. Yu, Z., An, B., Ramshaw, J. A. M. & Brodsky, B. Bacterial collagen-like proteins that form triple-helical structures. *J. Struct. Biol.* **186,** 451–461 (2014).
2. Hinman, M. B. & Lewis, R. V. Isolation of a clone encoding a second dragline silk fibroin. Nephila clavipes dragline silk is a two-protein fiber. *J. Biol. Chem.* **267,** 19320–19324 (1992).
3. Sponner, A. *et al.* The conserved C-termini contribute to the properties of spider silk fibroins. *Biochem. Biophys. Res. Commun.* **338,** 897–902 (2005).
4. Lane, A. K., Hayashi, C. Y., Whitworth, G. B. & Ayoub, N. A. Complex gene expression in the dragline silk producing glands of the Western black widow (Latrodectus hesperus). *BMC Genomics* **14,** 846 (2013).
5. Ayoub, N. A. & Hayashi, C. Y. Multiple recombining loci encode MaSp1, the primary constituent of dragline silk, in widow spiders (Latrodectus: Theridiidae). *Mol. Biol. Evol.* **25,** 277–286 (2008).
