# Supplementary material for: The transcriptome of Darwin’s bark spider silk glands predicts proteins contributing to dragline silk toughness
Source: Commun Biol. 2019 Jul 25;2:275. doi: 10.1038/s42003-019-0496-1 (PMC6658490; doi:10.1038/s42003-019-0496-1)
Supplement: Supplementary file 3 — Supplementary Data 10 [file 42003_2019_496_MOESM3_ESM.docx]

**Additional description of spidroin sequences**

**Longest protein sequences for each *C. darwini* spidroin C-terminal cluster.** These sequences were used in protein analyses. Name begins with name used in main text, figures and tables, followed by transcript translated from, those with names beginning with “c” derived from Iso-Seq assembly, those starting with “TR” derived from Illumina assembly. Name containing “RC” has coding sequence translated in reverse complement direction.

>MaSp2_c6135_f1p11_2123

YGPGGAAAAAAAAGGYGPGGSGPSGPGSQGPSGPGSQGPGGAGPYGPGGAAAAAAAAGGYGPGSQGPSGPGSQGPGGAGPYGPGGAAAAAAAAGGYAPAGQGQSGPGSQGQGQSGPGSQGPGGAGPYGPGGAAAAGGYGPGGQGPSGPGSQGPGGRGPSGPGGAAAAAASAYGPGGQGPSGPGSQGPGGQGPYGPGAAAAAAAAGGYGPGGRGPSGPGSQGPSGPGSQGPGGAGPYGPGGAAAAAAAAGGYAPAGQGQSGPGSQGQGQSGPGSQGPGGAGPYGPGGAAAAAAAAGGYGPGGQGPSGPGSQGPGSQGPSGPGLAAAAAAAGGYGPGGQGPSGSASQGPGGQGPYGPGGAAAAAAAAGGYGPGGSGPSGPGSQGPSGPGSQGPGGAGPYGPGGAAAAAAAAGGYGPGSQGPSGPGSQGPGGRGPSGPGSQGPGGAGPYGPGGAAAAAAAAGGYGPGSQGPSGPGSQGPGGQGPYGPGGAAAAAAAAGGYGPGSQGPSGPGSQGPGGQGPYGPSGAAAAAAAAGGYGPAGQGPSGPGSQGPGGQGPSGPGGYGPSSAAAAFGGYGPSGQIPSAAAAASRLSSPAVASRVSSTVSSLVSSGPTSQGALSNAISNAVSQISASNPGLSGCDVLVQALLEIVSALVHILGSSSVGQVSYNTAGQSAAVVSQSISQALG

>MaSp1a_c23000_f4p48_1723

AGGAGGRGGLGGQGGGQGAGGAGQGGYGSGLGGLGGGAAAAAAAAGGAGGLGGQGGGQGAGQGGYGSGQGGQGAGSAAAAAAAGGAGGRGGLGGQGGGQGAGGAGQGGYGSGLGGLGGGAAAAAAAAGGAGGLGGQGGGQGAGQGGYGSGQGGQGAGSAAAAAAAGGRGGYGGQGGGQGAGGAGQGGYGSGLGGLGGGAAAAAAAAGGAGGLGGQGGGQGAGQGGYGSGQGGQGAGSAAAAAAAGGAGGLGGYGGQGGGQGAGGAGQGGYGSGLGGLGGGAAAAAAAAGGAGGLVGQGGGQGAGQGGYGSGQGGQGAGSAAAAAAAGGAGGRGGLGGQGGGQGAGGAGQGGYGSGLGGLGGGASAAAAAAGGAGGLGGQGGGQGAGQGGYGSGQGGQGAGSAAAAAAAGGSGGLGGQGGYGGQGGYGGGYGGQQVAASATTASAAASRLSSPAASSRVSSAVSSLVSSGPTSPAALSNTISNVVSQVGASNPGLSGCDVLVQALLEIVSALIHILGSSSIGQVNYGATAQSTGIVSQSISQALG

>MaSp1b_c18326/f3p41/1509

QGGGQGAGQGGYGSGQGGQGAGSAAAAAAAGGRGGYGGQGGGQGAGGAGQGGYGSGLGGLGGGAAAAAGGAGGLGGQGGGQGAGQGGYGSGQGGQGAGSAAAAAAAGGRGGYGGQGGGQGAGGAGQGGYGSGLGGLGGGAAAAAAAAGGAGGLAGQGGGQGAGQGGYGSGQGGQGAGSAAAAAAAGGAGGRGGYGGQGGQGAGGAGQGGYGSGLGGLGGGAAAAAAAAGGAGGLGGQGGGQGAGQGGYGSGQGGQGAGSAAAAAAAGGAGGQGGYGGQGGQGAGGAGQGGYGSGLGGVGGGAAAGGAGGLGGQGGGQGAGQGGYGSGQGGQGAGSAAAASAAGGALGLGGQGGYGGQMGYGGGYGGQQVAASAATASAAASRLSSPDASSRVSSAVSSLVSSGPTNPAALSNTIGSVVSQIGASNPGLSGCDILVQALLEIVSALIQILASSSIGHVNYGATAQSTGIVSQSISQALG

>MaSp4a_c26805_f1p25_2132

PQSPYGPGPQGPGPQGPGPQGPSGPGPQRPQGPGPQGPYGPGGVSVVSATVSGPGPQGPSGPGPQGPYGPGPQGPGPQGPGPQLPGPQGPSGPGPQGPYGPGPQGPGPQGPGPQGPSGPGPQQPQGPGPQRPYGPGGVSVVSTTVSGPGPQGPLGPGAQVPYGPGPQVPGPQGPGPQGPSGPGPQRPQGPGPQGPYGPGGVSVVSQTVSGPGPQGPSGPGPQGPYGPGPQGPGPQGPAPQGPSGPGPQRPQGPGPQRPYGPGGISVVSTTVSGPGPQGPSAPGPQGPYGPGPQVPGPQGPGPQGPSGPGPQRPQGPGPQGPYGPGGVSVVSQTVSGPGPQGPSGPGPQGPYGPGPQGPGPQGPGPQGPSGAGPQRPQGPGPQGPYGPGGVSVVSATVSGPGPQGPSGPGPQGPYGPGPQGPGPQRPVPQGPSGPRPQQPQGPGPQRPFGPGGVSAVSTTVFGPGPQGPSGPGPQGPYGPGPQGPGPQGPGPQGPSGQGPQRPSGPRPQGPYGPGGISVVSATVSGPGPQGPSGPGPQRPYGPGPEGPGPQGAGPQGPGLQRPSGPGPQGPYGPGPRGPSSTPESAAINAASRLSSPAASSRVSSTVSQLVSSGPPNSAAVSGAISSLVSQVSASNPGLSGCDILVQALMELLSALVSIVGSSSIGQVNYGASGQYAQLVSQAIGQAF

>MaSp4b _c19205_f1p0_1636

PQGPYGPGPQGPGPQGPGPQGPGPQGPGPRGPSGPGPQGPYGPGGVSVVSASVSGPGPQGPSGPGPQGPYGPGPQGPGPQGPGPQGPGPQGPGPRGPSGPGPQGPYGPGGVSVASASVSGPGPQGPSGPGPQGPYGPGPQGPGPRGPGPQGPGPQGPGPQGPSGPGPQGPYGPGGVSVVSASVSGPGPQGPSGPGPQGPYGPGPQGPGPQGPGPQGPGPQGPGPRGPSGPGPQGPYGPGGVSVVSASVSGPGPQGPSGPGPQGPYGPGPQGPGPQVPGPQGPGPQGPGPQGPSGPGPQGPYGPGGVSVVSASVSGPGPQGPSGPGPQGPYGPGPQGPGPRGPGPQGPGPQGPGPQGPSGPGPQGPYGPGGVSVVSASVSGPGPQGPSGPAVNAAARLSSPDASSRVSSTVSQLVSGGPTSGAAVSNALSSLVSQVGASNPGLSGCDILVQALMEMLSALVSIVGSSSIGQVNYGASGQYTQMIGQAIAQAF

>MaSp1c_c31462_f1p21_1336

GRGGYGGRGGAGSSSAAAAAGSGGDGSGSGGYGGXGGQGGDGASSAAAAAGSGGDGSGSGGYGGRGGRGGQGGAGSSSAAAAAGSGGDGSGSGGYGGRGGRGGAGSSSAASAAAGGEDGFGRGGYGGRGGAGSSSAAAAAGSGGDGSGSGGYGGRGGQGGAGSSSAAAAGSGGDGYGSGGYGGRGGQGGAGSSSAASAAGSGGDGFGSGFYGGRGGEGGAGSSSAAAAAGSGGDGYGSGGYGGRGGQGGAGGASASAVAAGGGRGQGGYGGRGGQGGAGSSSASSTASAAASRLYSPDSSARISSAVSSLASYGPNNPTALSDVISNTMSQVSYSSPELSGCDVLVQTLMEVVSALVHILSVSDIGPVAYDSDQAVQVVGQSFNNLMY

>MaSp5_c20015_f4p27_1444

GGLGDSGGGLGGSRGGLGGSGGGLGGSGGGLGGSGGGLGGSGGGLGGSGGGQGGSEGGLGGSGGGLGGSSGRLGGSGGRLGGSGGGLGRSGGGIGGSGGGLGGSGRGLGGSGVGPGGSGGGLGDSGGGLGGSGGGVGGSGGGLGDSGGRLGSSGSVGGSGGRGGLGGPGSSGGTDGQGAMGGSGGRGLDGPGSLGGTGGQGGMDGPGGGVDGDYSAASASSRGLGGSGPGGYGRSLGGPGGFGGDRDLGDSASSAAASAGGDGGSSGPGKRGGYGRGSGGAKGLSGSGGGIGSGAAATLAGGLGGSLSAGSGEFLGTSGGRGGDSSQTSASSTISSAASRLSSPEASSRISSVVSSFLSNGIDNPSSLSSSLSGIVSRISSLNPMLSSCDILLQALLEIVSALLQILASSNIGPIDYSSTRQSTGIVSQSVYQAFS

>MiSp1_TR41386_c0_g1_i1

GAGGAGGRGGYGGGSGAGAGAAAAAGAGAGGGYGGGQGGYGGGSGSAAGGASAAGAAVGSYGSGGYGGGASYASSSAGSVVNTVSSRITSSESSSRISSAASTLTAGGALNAAALSDVIGNVYSQVSASAGGASGAEVLVQTLLEIVSALLHILSSSNIGYVDFGGVSSSANAVAQSVAAALG

>Flag_RC_TR56526_c0_g1_i1 length=1619

ALGSGGGFRGFGGPGGPGGPGGPGAPGAPGGPGVGGPGGYYGPGAGGAGGMLGSGAGGVSGGPGGLGGPGGFGGPGGVGGLGGMGGVGPGGSGIMYGPGAGGAGGGFGSGAGGAAGGQGGFGGAGGPGGPGGAGGPGAAGGVGGISGPGGAGPSGGGAGGVTVVDNLSVNVGGAGAGGAGTGGAGGSLGGLGGFGGPGGPGGPGGPGGPGGSGAAGGMTGPGAGGSAGGAGGSGPITISGTLSVGGAGAGGAGPGAGGRYGSGGSGGGAGGFGGPGGPGGPGGPGGPGGLGGAGAGGVGPGTGGASSPGGGSGPVTVTDNVSVTVGGSGGSGGSDAGGAGGALGSGGGFRGFGGPGGPGGPGGPGAPGAPGGAGAGIIGGAPSPSGGSSGPVTVSDNISITIGGQMSSAGSAGPGFQGQPVVSRLPSLVNGMLGSMQANGLNYQNLGNLLSRYSTGSGTCNSNDLNLLMEALQAALHCLSYPGPASVPSMPPPSSTSAYMQSIRRVFGY

>TuSp1_TR23580_c0_g2_i2 length=1093

AASEAASSSASSSSASAFAQSASQSLAMSSSFASAFSAAASSAESLRSLGFQIGNALVNNLGLRLQPADVAQALSAVGTGASTNAYANALASAVARAAASQGTLNAGNSGSLASAASAAISAAAASASSSQFQSAAAQQQAAAAAFRQAASQSGSQSAARSGSQSSSSATTTSTSGSQAASEAASRRAGASAGAGSNAGAFGAAFGRSAGGTGANAGAFGAALGRPVGQDVAPSLQSALAPVLSSSVLSSSDATARVNSLAESVSSAIASSGGSLNVATFLDSLSSVGSQVRSGTSLDASQATSEVLLEAIAALIKVINGAGITRVNLDNVSNVNSALVSALAG

>MiSp2_RC_TR78167_c0_g3_i1 length=925

GYGGGAGAGAGAAGAGGAGGYGGGYGGGAGAGAGAAGAGGAGGYGGGYGGGAGAGAAAGAGAGGRGVYGGGSGAGAAAGAGAGAAVGYGGGYGGGAGAGAGAAAAAGAGAGAGGGYGGYSGGAGAGAGAAAAAGAGAGGSYGGGFGGYSVGAGSGAGVVSTVSSTTTRLSSAEASSRISTAASSLVAGGVVNTSVLPSVISNLYSQVSASSPGASSSEVLVQVLLEIISSLIHILGSSSVGQINLGSVASSAAAVGQSLQAVMG

>PySp_TR73277|c0_g1_i1

SRAPSAYSGGSMASLPSQGSSAFSGGSVASLQSQGSSAYSGASAASLQSRASSAYSGGSARSLQSQASSAFSGGSGTQYGSSQSSSMSSSAALGPIIAPVSVPSYVQPTSRPASVGISSGSSLAVSSQQLMSPAAAQRISALSNSLASAIAGGRINYGALSNSLAAASRQIQSGSGMSKTEAIVETLLETLAALLETISGSSGGGQTAQMLLQALA

>AcSp_TR33034|c0_g1_i2

VCANVIVSACVKAMLSSGVSVSDSNSQQIASQLSSTIVDAVCGAAGRAGMRIPDSVVQSDKNLVSQTITSISSTSSATTTTTTSVQSTDSSSSSFSGLDSTGGYTGIPSGGYPGGPDYGQGSDNVQRQLVQTLTEALQGTQSMSLVSRAKLFSINSSYRTDFARLVSGPMNLGGSAQSELLVSLAGISPNSDARVCANVIVSACVKAMLSSGVSVSDSNSQQIASQLSSTIVDAVCGAAGRAGMRIPDSVVQSDKNLVSQTISSISSTSSATTTTTTSVQSTDSSSSSFSGLDSTGGYTGIPSGGYPGGPDYGQGSDNVQRQLVQTLTEALQGTQSMKLVFGAQLFPNNSFRTEFARLVSGSMNLGGSASSELLVSLAGISPTSDARVSFKVLVSACVGAMLSSGVSVSDSNCQQIASQLSSTIVDAVCGAAGRAGVRIPDSVVQSDKNLVSQTISSTSSATTTSATSVQSTDLSSSSFLGQDSTGVSSSTSVSSIINSPNGLKSPQANARINSLASLFNNAIGSNGVQIDAVSQGLAGIMSNLKRSGMSPTQAQVEALVEMNCALLKIVVASQGGSPNSVSSSSMTSLLSVML

>AgSp_TR66083|c0_g1_i1

KFIVPNGAFSTPGSIPGPDGKPIHVQPAGPGTTPGAITDSDGDVVQIFLPSTPAPKPVNPTTPTAITGPKGNPIVIYPAGPGTTPGTVTGPDGKPTQFIVPLGAFSTPGSIPGPDGKPIPVEPAGPGTTPGTLTDPDGRVNRIYLPTTPAPPSYQPQVPLTTTPIPGPGPQPIQIIPAGPGTTPGTVTGPDGRPTQFIVPQGAFSTPGTIPGADGKPIPVEPAGPGMTPGAQTGPDGKITRIVLPTTTPLPPPPGPLNPDGLPVAPFGPGNSPNYQSPGGYPGFQFPGYPGAPGSDGPTRYINSNELPSGETPDGYLNVDSLPDFVTPGFPQSPLGYLDFSQLPDNYSPDFPGQLVFPGYPNSPGNGRNTPGGFLSFPDFPKDITNKLNSPFSFPQIIQALQPLFPGNTINMGAIPKDQLQNIPGLDGDYNNLQIPDMGDSSHPTGGVFYLPELIRLISYLPVGSFPGRGPGTMNPDGTYSDPFDFPGLNGAPGYICDYPDNGDATPDLGQEVQGTNQGPVGDVEDAAPGSDDDLGAPAPQLESDESDCDDDVFGTFNKARSSLLDVASSTGVQTISDLMQALISGINPYENTVDYNDFFNELSSLFSQVRAGSDSQGPNKEFIKILFEALVASLEALNAAKVNGFRDVSVPSALPVYTSFLS

***N terminal clusters***

Longest exemplars per cluster containing N-termini (Sequences contain no-C-terminus)

>MaSp4a_RC_TR60988_c2_g1_i1

MSYLTRLALALLAVLSTQAIFANGQITPWSNTRLAEAFINSFMSKVGYSGAFTAEQMDDMSTVSDTIMTAMDKMASSNKSSKSKLQALNMAFASTMAEIAATEEGGQSMAVKTNAITDALSAAFLETTGQVNYQFINEIKSLVYMLAQQSMNDVYASAGTASGGGYGPGPQGPSGPGPYGPRGVSVVSTSVSGPGPQGPSGPGPQGPYGPGPQGPGPQGPGPQGPGPQGP

>MaSp2_RC_TR18301_c2_g3_i1

MYSSTRLALTLLAVLCTQAIFTAAQAPSPWESTALAERFMASFLAATGQSGAFTAEQLDDMSTIGDTLSSAMDKMARSNKSSKSKLQALNMAFASSMAEIAAVEQGGQSIAVKTNAIENALISAFMQTTGAVNYQFVSEIRNLVNMMAQASANEVSYASAGGSAAASASGGYGPSSQGPSGPGGYSSSVSVSGVYGPGPQGPAPQGPSGPTPQGPQGISSSVSVSGVYGPGPQGPAPQGPSGPTPQGPQGTYSSVSVSGAYGPGPQGPAGQGPSGPGPQGPGGAAAAAAAASGYGPGGQGPSGSGGQGPSGPGGS

>Fibroin1_MaSp1-like_N-term_TR44210_c2_g2_i1

MTWTSRLALSLLVAICTQSMFALGQDNTPWSSTGTAESFMSSFMSAAGNSGAFTADQLDDMNTITDTIRSAMDKMARSNKSSKSKLQALNMAFSSAMAEIAIDEGGQSVGYKTDAIADALSQAFLQTTGVVNGAFINEIRSLISMFAQNSANAIGSGGSSASVSVAASAGGGYGGQGSYGPGPQGPSGPGPQGAGASSASAVSAASGPGGYAPGPQGPSGPQGPGQSSYQYSVSISTQGGSQGGYGGQQGGAGQGGYGGGLGGQGAGAAAAAAAAGGAGGLGGQGGQGGGQGAGGAGQGGYGSGLGGQGGGAAAAAAAAGGAGGLGGQGGQGGGQGAGGAGQGGYGSGLGGQGGGAAAAAAAAGG

>Fibroin2 _MaSp5-like_N-term_TR55725_c2_g1_i8

MSWTSRHALFYLLVICTQSVLALGRNNNPWSNPSSAESFMNYFMDGVTNSGSFTPDQLDDMCVICDTIKATTDRMSRSNKNTESSLQALNIAFASAVAEIAAAEGTENIGMKTGAITDALSSAFMQTTGQVNTEFVNEIRSLINMFSQVSRNNISQGGLGGTGDVGGAGGRGGLSGAGGLSGPSGLGGTGGRGGFAGSGGGLGASASSASSGGPGGSGQGGYGGSLGGPGGFGRSGGGLGGGDSAASTSSIGSGGPGGSGQGGYGGSLGGPGGFGRSGGGLGGGDSAASASSVGSGGPDGSGPGGYRGNIDGPGGFGRSGGGLGGGDSAASASSVGSGGPDGSGPGGYGGSLGGPGGFGRSGGGLGG

>Fibroin3_MaSp1-like_Nterm_TR67764_c0_g1_i1

MIWIARLALLVAVAFSTQSQLALGQDNTPWSSTSSAERFMEAFIGGAQNTGVFTDGQISDMKDIIDTIKAAMEKMKNKNKNSKSVLQALNMAFASSVSEIAVTEGSQSIEAKTNAISDALASAFMQTTGSVNQGFINEMKTLVSMFAQTSFNDVSYSDSSASSSSGGYGSPGGYNSGGPGAASAVSTSSASGAAEPIFYGQGPSAYQYSVSISTQSGGQGGYEGIGGVGTASAASAGGGTGAGESGQGGYGGIGTGSSSAAAAGAGGAGGFGPGGYGMGGLGGAGSAAAAAGGAGGIGPGGYGGNGGQGGVGSASAAAAGAGGTGGFGPGGYGGGGLGGEGAASAAAAGAGGAGGFGHAAGAGGPDGYGSGGYGGRGGQGGAG

>PySp_Nterm_TR12136|c0_g1_i1_len=1230

MSWLPALSLLLVLLVPNTNAISASKASFQDEGTTIYLLRSILEYLRECDVLKIDQESDAVNALFEVSLLFQNNVKMSKRKQAIASKLAGIIMEGLEGSNETAYKLDCATNAMIAAMENTSGTVDMSFIDSVKELAVVMYNNDIEAKLEELEEEQEELYQEQLLSSVIPESQISTDQSDYGSIQIQNQLVDQGVSSITTSNENQIQTQQSVTNQIQLTSTAETQSQQSAGTTASQQSYIDGQQSYISQQQSAESQQQYANTQQQSVESQQQSAESQQQYANSQQQATGSTQNYATSQQNIQSSNSYEDQSSISQAQEVQSSYSQNQYSASHQQATDTLQQTIESQPQYTSSQQQIQQSSNEYSDQSSLTQYQVDSSSASIYFQTDVVHNRVAQSLLSSSVL

Longest “short isoforms” per type including N and C-terminal domains (see Supplementary Notes)

>MaSp2_c24257_f1p13_1860_ Nterm_Cterm length=593

MYSSTRLALTLLAVLCTQAIFTAAQAPSPWESTALAERFMASFLAATGQSGAFTAEQLDDMSTIGDTLSSAMDKMARSNKSSKSKLQALNMAFASSMAEIAAVEQGGQSIAVKTNAIENALISAFMQTTGAVNYQFVSEIRNLVNMMAQASANEVSYASAGGSAAASASGGYGPSSQGPSGPGGYSSSVSVSGVYGPGPQGPAPQGPSGPTPQGPQGISSSVSVSGVYGPGPQGPAPQGPSGPTPQGPQGTYSSVSVSGAYGPGPQGPAGQGPSGPGPQGPGGAAAAAAAASGYGPGGQGPSGPGSQGPGGQGPSGPGSQGPGGQGPYGPGGAAAAAAATGGYGQGGYGSGQGGQGAGSAAAAAAAGGAGGRGGYGGQGGQGAGGAGQGGYGSGLGGLGGGAAAAAAAAGGAGGLGGQGGGQGAGQGGYGSGQGGQGAGSAAAAAAAGGAGGRGGLGGQGAGQGGYGSGQGGYGPSGQIPSAAAAASRLSSPAVASRVSSTVSSLVSSGPTSQGALSNAISNAVSQISASNPGLSGCDVLVQALLEIVSALVHILGSSSVGQVSYNTAGQSAAVVSQSISQALG

>MaSp4a_c16597_f1p7_1747_Nterm_Cterm length=504

MSYLTRLALALLAVLSTQAIFANGQITPWSNTRLAEAFINSFMSKVGYSGAFTAEQMDDMSTVSDTIMTAMDKMASSNKSSKSKLQALNMAFASTMAEIAATEEGGQSMAVKTNAITDALSAAFLETTGQVNYQFINEIKSLVYMLAQQSMNDVYASAGTASGGGYGPGPQGPSGPGPYGPRGVSVVSTSVSGPGPQGPSGPGPQGPYGPGPQGPGPQGPGPQGPGPQGPSGPGPQGPYGPGSVSVVSGSVSGPGPQGSSGPGPQGPYGPGPQGPAPKGPGPQGPGPQGSGPQGPSGPGPQGPYGPGGVSVVSTTVSGPGPSGPGPQGPYGPAPQGPGPQGPGPQGPGPQGPGPQGPSGQGPQRPSGPRPQGPYGPGGISVVSATVSGPGPQGPSGPGPQRPYGPGPEGPGPQGAGPQGPGLQRPSGPGPQGPYGPGPRGPSSTPESAAINAASRLSSPAASSRVSSTVSQLVSSGPPNSAAVSGAISS

>MaSp4b_c13621_f1p2_1363_ Nterm_Cterm length=401_ Nterm

MSYLTRLALALLAVLSTQAIFANGQNPWSNTGLAEAFINSFMSKVGYSGAFTADQMDDMSTVSDTIMSAMDKMARSNKSSKSKLQALNMAFASTMAEIAATEEGGQSMSVKTNAITDALSAAFLETTGQVNYQFINEIKSLVYMLAQQSMNDVYASAGTASGGGYGPGPQGPSAPGPYGPGGVSVVSASVSGPGPQGPSGPGPQGPYGPGPQGPAPQGPGPQGPSGPGPQGPYGPGPQGPGPRGPGPQGPGPQGPGPQGPSGPGPQGPYGPGGVSVVSASVSGPGPQGPSGPAVNAAARLSSPDASSRVSSTVSQLVSGGPTSGAAVSNALSSLVSQVGASNPGLSGCDILVQALMEMLSALVSIVGSSSIGQVNYGASGQYTQMIGQAIAQAF

**Additional long Piriform spidroin lacking N and C-terminal domains (only repetitive sequence) identified from blast annotations in Illumina transcriptomes**

>PySp TR43065|c0_g1_i1 len=3005

QSSNTQSQQTATSQSQQSSFSQSSQQAYNAASTAASSVQQSVTNTFNSQTVQNSVAQSLMSSSVLNTIASGQTSATQSDFASVISNALANTLGVSQSSVNGVVNQQISNLRPGTSASTFAQTVSNIISSLLPQSNAAVAGQEQSVSQSVSASVVNALANLISRQSRPVALPQPAPRPIPAPRPQPAPRPLPAPRPVAPVIQAPAPVVSQIQQSSNTQSQQSSISQSQQTAFAESQQRGISESQISSFSTSQQTGNLYGSATTATSNVVQAPAVISRTANYFNSELARNSLLSYLQSSDILNVLASGQTSTSLSDLSSVIANSVAKVLGGSQTTFYNVINQQLSSVGSGSSAAALAQAVADSVSGLLENSGVAIAGREQDISSSISSSILSALAQRVSQSSIQAPAPIARPLSAPRPVSAPSFAPQPVVSQSQQSFASQINSQQSAASQTQQSVAQAQQSSVAQVQQSSYGQQQSTYAQAQQFSSGQSQQASNAYSTASVGPSNVAQSPSLSSSGYFNSQIVQNNLVSSLQSSNAFNSVIYGQTGASLADVESAIASSISQSIGIPLSSVQNYIRQQLSGLGSGVTTSTFAQAVANGVSNMIQNSGVASAGQEQNVSQIIFSGIQTALTGLMSQRSRQLPAPLPTPSQPISPSVYAPQPAISPIQQSFATQAQSQRSSVGQSQQSSFAQSQQISAQSQQAKSQQSSFGQSQQSRYAQAQQVSSQPQQSTLYGQSQQSSNLYSTGSSSSSGLTQSSSYFNPEIVQNNLVSSLQSSSALNSIASGQISASTSDISSVIANAVAPLAGLSQSVVQNVINQQLSSVGSGAPAGIFARALATSLSRLIQKSGIASAGEESGISQMISSGIESALIKLVSQKSMFAPAPRNAPMPISQPAPLPQTVPAYAPRPSPVYFTAPSLQQSSISQNQQSNNAYSSNAAAQNQFQQASSLQSQASSAYSGGTAATLQSQGSSAYSGGSMASLQSQGSSAYSGGSMASLQSQGSS
