# Supplementary material for: The transcriptome of Darwin’s bark spider silk glands predicts proteins contributing to dragline silk toughness
Source: Commun Biol. 2019 Jul 25;2:275. doi: 10.1038/s42003-019-0496-1 (PMC6658490; doi:10.1038/s42003-019-0496-1)
Supplement: Supplementary file 4 — Description of additional supplementary items [file 42003_2019_496_MOESM4_ESM.docx]

**Description of Additional Supplementary Files**

File Name: **Supplementary Data 1**

Description: Contains a summary of spidroins identified from *Caerostris darwini* Major Ampullate Silk Glands.

File Name: **Supplementary Data 2**

Description: Contains amino acid composition data for *Caerostris darwini* dragline and Major ampullate (MA) glands in comparison to dragline from other araneoid species.

File Name: **Supplementary Data 3**

Description: Contains amino acid composition data for *Caerostris darwini* spidroins expressed in percentages.

File Name: **Supplementary Data 4**

Description: Contains percentage of abundant amino acids in *Caerostris darwini* (Cd) and araneoid spidroins.

File Name: **Supplementary Data 5**

Description: Contains predicted secondary structures for *Caerostris darwini* (Cd) and select araneoid spidroins.

File Name: **Supplementary Data 6**

Description: Contains top forty most expressed transcripts from Illumina de novo *C. darwini* Major Ampullate Silk Gland Transcriptome.

File Name: **Supplementary Data 7**

Description: Contains expression data for *C. darwini* spidroins in major ampullate glands in TPM using last 500 bp.

File Name: **Supplementary Data 8**

Description: Contains descriptors of additional spidroin sequences used in phylogenetic analyses.

File Name: **Supplementary Data 9**

Description: Contains description of oligonucleotide sequences trimmed from raw RNA-Seq reads.

File Name: **Supplementary Data 10**

Description: Contains additional descriptions of spidroin sequences analyzed in this study, including representatives of longest spidroins.
