# Supplementary material for: The transcriptome of Darwin’s bark spider silk glands predicts proteins contributing to dragline silk toughness
Source: Commun Biol. 2019 Jul 25;2:275. doi: 10.1038/s42003-019-0496-1 (PMC6658490; doi:10.1038/s42003-019-0496-1)
Supplement: Supplementary file 5 — Reporting Summary [file 42003_2019_496_MOESM5_ESM.pdf]

nature research

Corresponding author(s): Jessica Garb

Last updated by author(s): May 21, 2019

Reporting Summary

Nature Research wishes to improve the reproducibility of the work that we publish. This form provides structure for consistency and transparency in reporting. For further information on Nature Research policies, see Authors & Referees and the Editorial Policy Checklist.

Statistics

For all statistical analyses, confirm that the following items are present in the figure legend, table legend, main text, or Methods section.

n/a	Confirmed	
☐	☒	The exact sample size (*n*) for each experimental group/condition, given as a discrete number and unit of measurement
☐	☒	A statement on whether measurements were taken from distinct samples or whether the same sample was measured repeatedly
☐	☒	The statistical test(s) used AND whether they are one- or two-sided<br>*Only common tests should be described solely by name; describe more complex techniques in the Methods section.*
☒	☐	A description of all covariates tested
☒	☐	A description of any assumptions or corrections, such as tests of normality and adjustment for multiple comparisons
☐	☒	A full description of the statistical parameters including central tendency (e.g. means) or other basic estimates (e.g. regression coefficient) AND variation (e.g. standard deviation) or associated estimates of uncertainty (e.g. confidence intervals)
☐	☒	For null hypothesis testing, the test statistic (e.g. *F*, *t*, *r*) with confidence intervals, effect sizes, degrees of freedom and *P* value noted<br>*Give P values as exact values whenever suitable.*
☐	☒	For Bayesian analysis, information on the choice of priors and Markov chain Monte Carlo settings
☒	☐	For hierarchical and complex designs, identification of the appropriate level for tests and full reporting of outcomes
☒	☐	Estimates of effect sizes (e.g. Cohen's *d*, Pearson's *r*), indicating how they were calculated

Our web collection on statistics for biologists contains articles on many of the points above.

Software and code

Policy information about availability of computer code

Data collection	Zen 2.3; ImageJ 1.50i ; Pacific Biosciences SMRT Analysis Pipeline 2.3.0; CUTADAPT 1.14; Trinity 2.0.6
Data analysis	BUSCO 2.0; blast 2.2.29+; Diamond 0.8.23; CD-HIT 4.6.4; EMBOSS 6.6.0 (getORF); MUSCLE v. 3.8.31; Genious 9.1.8 (EMBOSS garnier plugin); Salmon v0.8.2; Mr. Bayes v. 3.2.6; ProtParam (http://web.expasy.org/protparam); Prism 7

For manuscripts utilizing custom algorithms or software that are central to the research but not yet described in published literature, software must be made available to editors/reviewers. We strongly encourage code deposition in a community repository (e.g. GitHub). See the Nature Research guidelines for submitting code & software for further information.

Data

Policy information about availability of data

All manuscripts must include a data availability statement. This statement should provide the following information, where applicable:

- Accession codes, unique identifiers, or web links for publicly available datasets
- A list of figures that have associated raw data
- A description of any restrictions on data availability

Sequence data from this study is available at NCBI's SRA database under the accession submissions SRR7499252, SRR7499250, SRR7499251. Assembled transcriptomes are available at NCBI's TSA database under accession numbers GGUO00000000 and GGTX00000000. Raw data associated with Figures 1-3 are in the Supplementary Data 1-9 and Supplementary Data 10 files.

Field-specific reporting

Please select the one below that is the best fit for your research. If you are not sure, read the appropriate sections before making your selection.

☒ Life sciences ☐ Behavioural & social sciences ☐ Ecological, evolutionary & environmental sciences

For a reference copy of the document with all sections, see nature.com/documents/nr-reporting-summary-flat.pdf

Life sciences study design

All studies must disclose on these points even when the disclosure is negative.

Sample size	Sample sizes were not pre-determined and were based on similar studies while balancing limited quantities of sample materials. Illumina RNA-Seq data was performed on two biological replicates, silk amino acid composition analyses used 3 biological replicates, measurement of gland morphology used 3-7 individuals per species. Generation of the Pacific Biosciences Iso-Seq transcripts was generated from one of the same individuals sampled for Illumina RNA-Seq enabling the comparison of independent sequencing techniques .
Data exclusions	Illumina reads with low quality scores were trimmed/removed prior to use in analyses.
Replication	Technical replicates were not performed; biological replicates were sampled as described in the sample size field. We sequenced silk gland cDNA transcripts using different sequencing methods: Pacific Biosciences Single Molecule Real Time (SMRT) sequencing of a single individual and Illumina RNA-Seq sequencing conducted on two biological samples. The individual used in SMRT sequencing was also used for one of the replicate RNA-Seq libraries, as described in the manuscript.
Randomization	Randomization was not performed in this study, as experiments were not part of this study.
Blinding	Blinding was not performed as part of this study, as experiments were not part of this study.

Reporting for specific materials, systems and methods

We require information from authors about some types of materials, experimental systems and methods used in many studies. Here, indicate whether each material, system or method listed is relevant to your study. If you are not sure if a list item applies to your research, read the appropriate section before selecting a response.

Materials & experimental systems

n/a	Involved in the study	
☒	☐	Antibodies
☒	☐	Eukaryotic cell lines
☒	☐	Palaeontology
☒	☐	Animals and other organisms
☒	☐	Human research participants
☒	☐	Clinical data

Methods

n/a	Involved in the study	
☒	☐	ChIP-seq
☒	☐	Flow cytometry
☒	☐	MRI-based neuroimaging
